# Intelligent Sensing Using Multiple Sensors for Material Characterization

**DOI:** 10.3390/s19214766

**Published:** 2019-11-02

**Authors:** Ali M. Albishi, Seyed H. Mirjahanmardi, Abdulbaset M. Ali, Vahid Nayyeri, Saud M. Wasly, Omar M. Ramahi

**Affiliations:** 1 Department of Electrical Engineering, King Saud University, Riyadh 11451, Saudi Arabia; aalbishi@KSU.EDU.SA; 2 Department of Electrical and Computer Engineering, University of Waterloo, Waterloo, ON N2L 3G1, Canada; shmirjahanmardi@uwaterloo.ca; 3 Faculty of Applied Science, University of British Columbia, Kelowna, BC V1V 1V7, Canada; abdulbaset.ali@ubc.ca; 4 School of Advanced Technologies, Iran University of Science and Technology, Tehran 1684613114, Iran; 5 Department of Electrical and Computer Engineering, King Abdulaziz University, Jeddah 21589, Saudi Arabia; swasly@kau.edu.sa

**Keywords:** artificial intelligence, complementary split-ring resonators, electrically-small resonators, fluid characterization, material measurements, neural networks, sensors

## Abstract

This paper presents a concept of an intelligent sensing technique based on modulating the frequency responses of microwave near-field sensors to characterize material parameters. The concept is based on the assumption that the physical parameters being extracted such as fluid concentration are constant over the range of frequency of the sensor. The modulation of the frequency response is based on the interactions between the material under test and multiple sensors. The concept is based on observing the responses of the sensors over a frequency wideband as vectors of many dimensions. The dimensions are then considered as the features for a neural network. With small datasets, the neural networks can produce highly accurate and generalized models. The concept is demonstrated by designing a microwave sensing system based on a two-port microstrip line exciting three-identical planar resonators. For experimental validation, the sensor is used to detect the concentration of a fluid material composed of two pure fluids. Very high accuracy is achieved.

## 1. Introduction

Modern technologies such as lap-on-a-chip involve fluidic and microfluidic analysis that require highly accurate sensing systems. These technologies can be implemented in many applications including environmental monitoring, agriculture, aquaculture, biotechnology, food production, and public health and safety [1]. The sensing systems need to be highly sensitive and selective, provide a very-fast response, miniaturized, and inexpensive to be suitable for real-life and in-the-field applications. Microwave sensors based on planar electrically-small resonators can offer many features for designing the sensing systems meeting these desirable requirements.

In electrically-small resonators, the physical dimensions of resonators are electrically small compared to the operating wavelength where the electromagnetic (EM) fields are enhanced in small areas [2]. By disturbing such field with the materials under test (MUT), the resonator can then be utilized to design different sensing modalities [3,4]. Compared to non-resonant sensors such as open-ended coaxial cables that are based on measuring the reflection coefficient, sensors based on resonators are less sensitive to the signal-to-noise ratio since the sensing mechanism is based on detecting the shifts in the resonance frequencies [5].

Planar resonators are unbounded structures, which makes their analytical models difficult to develop for characterizing the MUT. However, as long as the sensing mechanism is based on measuring the changes in the resonance frequencies, full-wave numerical simulations can be utilized to model the responses of the resonators as functions of the properties of the MUT (e.g., dielectric constant). By using fitting function techniques, the models can then be constructed [6,7,8]. Nevertheless, since the sensing mechanism is based *only* on a single or multiple resonance frequencies, the utilization of the resonators in the sensing systems are still at the preliminary stage. This is due to the limitations and difficulties in constructing either analytical models or models based on numerical results at multiple frequencies.

In this paper, we introduce a concept for designing microwave intelligent sensing (MIS) systems based on modulating the frequency response of multiple electrically-small resonators with the MUT. The sensing mechanism of the systems is based on measuring the changes of the transmission coefficients of microstrip lines. When multiple resonators are used, coupling to the resonators will increase the bandwidth of the bandgap. Thus, if one of the resonators is loaded with the material under test whereas the other resonators are loaded with well reference materials, the coupling between the resonators will be affected and that will add more information about the MUT to the response, which is the main idea of this work. The concept is based on the assumption that the targeted physical parameters such as fluid concentration are constant over the range of frequency of the sensor. The modulation is based on the interactions between the sensors and the MUT. We show that using many points at different frequencies can reduce the need for a large number of MUT samples to achieve higher accurate neural networks. For validation, an MIS is developed based on utilizing three-identical resonators. The concept was tested using numerical simulation and verified experimentally to characterize the concentration of a fluid mixture.

## 2. The MIS Based on Multiple Resonators

In practical sensing applications, the accuracy of the extracted results can be degraded due to many sources of errors including air gaps between the sensor and the MUT and the background noise. Thus, for characterizing a single physical parameter, such as fluid concentrations or the permittivity or permeability, using the information provided by a single frequency point or the shifts in the resonance frequencies are not sufficient to meet the need for highly reliable sensing systems. If the sensing systems are going to be utilized for characterizing many physical parameters simultaneously, the limitations of models (developed using numerical simulations and fitting function techniques) increase. Of course, the sensing mechanism based on measuring only the shifts in the resonance frequencies or the reflection coefficients at a single frequency will make the models’ construction process easier; however, these models will limit the sensitivity due to measurements’ errors or the background noise.

To overcome these limitations, the sensing systems can be designed by utilizing electrically-small resonators where the response over many frequencies can be observed. Microwave planar electrically-small resonators that are based on two-port microstrip line systems terminated by load impedances have an important feature compared to one-port resonant systems such as open-ended coaxial resonator probes and evanescent microwave probes [9,10]. In a two-port system, the MUT affects the resonators but not the terminations (the loads). Hence, the system does not require matching networks to observe the response. For instance, in a 50 Ω system, one designs a microstrip line on a substrate to have a 50 Ω characteristic impedance over a range of frequencies. Then, the system is coupled to a planar resonator and terminated by 50 Ω load impedance. Therefore, observing the transmission coefficient will reveal that the system exhibits a bandgap over a certain range of frequencies. The bandgap is a function of many parameters such as the topology of the resonator and its physical dimensions, the coupling between the line and the resonator, the substrate, and the surrounding environment. Since the sensing system is not enclosed by a perfectly conducting surface, the system will exchange energy with the surrounding environment. The quality of that exchange will determine the system sensitivity, which is limited by the bandgap’s characteristic caused by the interaction between the MUT, the resonators, and the environment. Therefore, having multiple resonators is expected to increase the sensitivity of the system over a broader bandwidth of the bandgap where each resonator can be modulated with different materials. In fact, some of the resonators can be used to modulate the response with a priori characterized materials (i.e., reference materials).

**Figure 1:**
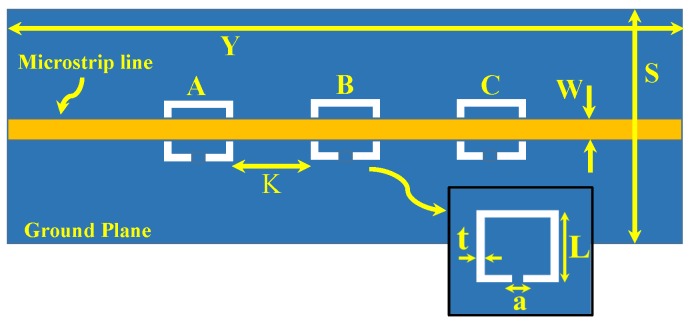
A two-port microstrip line exciting three identical complementary split-ring resonators (CSRRs) (A, B, and C) that are etched out in the ground plane of a microstrip transmission line.

Electrically-small resonators such as complementary split-ring resonators (CSRRs) show higher sensitivity to detect the presence of the dielectric materials compared to split-ring resonators (SRRs) [6]. Figure 1 shows an MIS based on a two-port microstrip line exciting three-identical CSRRs (A, B, and C). Although the MIS is based on three-identical CSRRs, the concept can be generalized to any number of identical or non-identical resonators.

The concept is demonstrated by characterizing the concentration of liquids. For instance, suppose that the MUT ($M_{m}$) is a mixture of two pure materials ($M_{l}$ and $M_{u}$), where $M_{u}$ has a dielectric constant higher than $M_{l}$. By loading the resonators A and C with $M_{u}$ and $M_{l}$ and resonator B with $M_{m}$, it is expected to have an intermediate resonance frequency between the resonance frequencies corresponding to $M_{u}$ and $M_{l}$, respectively. The transmission coefficient can be considered as a vector of many components, $|S_{21}\left(f_{1},f_{2},\ldots ,f_{n}\right)|$. The upper and lower resonance frequencies of $M_{l}$ and $M_{u}$ directly depend on their dielectric constants.

The primary objective of the supervised learning algorithms using neural networks is to find the relationship between the input, which is the vector $|S_{21}\left(f_{1},f_{2},\ldots ,f_{n}\right)|$, and the output, which is the fluid’s concentration. The outcome of the neural network can be depicted in a one-dimensional visualization, where the abscissa represents the input (the vector of $|S_{21}\left(f_{1},f_{2},\ldots ,f_{n}\right)|$) and the ordinate represents the output (the concentration).

## 3. The MIS Design and Validation

**Figure 2:**
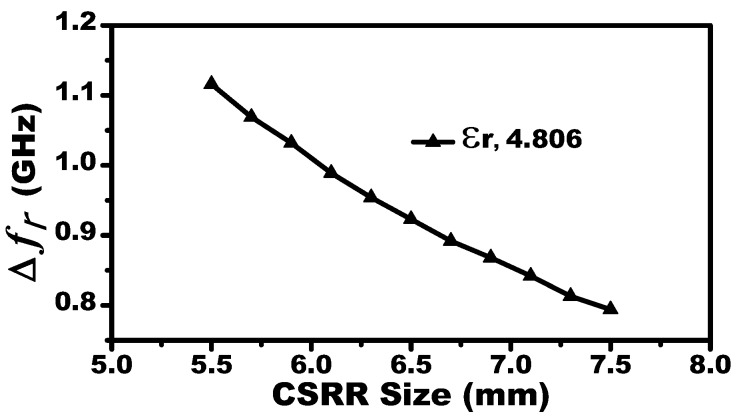
The sensitivity of a single CSRR (represented by a shift in the resonance frequency) as a function of the CSRR’s dimension when loaded with a dielectric slab having a relative permittivity of 4.8.

**Figure 3:**
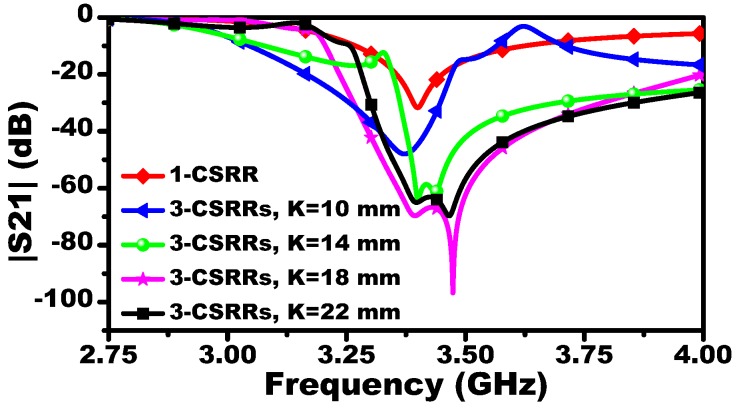
The scattering parameter $|S_{21}|$ of a single CSRR and three identical-coupled CSRRs with different values of K.

In this work, a two-port microstrip line is used to excite the resonators. Since the response of the MIS ($|S_{21}|$ dB) will be observed by a 50 Ω vector network analyzer (VNA), the microstrip line with a 50 Ω characteristic impedance is designed (line width of 1.63 mm, line thickness of W = 0.75 mm, Rogers RO4350 substrate with relative permittivity of 3.66, and a loss tangent of 0.0031). The sensitivity of the systems is proportional to the resonators’ lengths. This can be confirmed by investigating the shifts in the resonance frequencies versus the dimension of a single CSRR-based sensor when the MUT is a slab of relative permittivity of 4.8 (see Figure 2). For a system designed to operate at a microwave frequency regime suitable for our measurement setup and taking into account the resonance frequency as a function of the resonators’ lengths, the dimensions of the CSRRs are L = 7.5 mm and t = a = 0.5 mm. The MIS system with different values of K is presented in Figure 3. For K = 10 mm, the MIS system exhibits a resonance frequency of 3.4 GHz.

**Figure 4:**
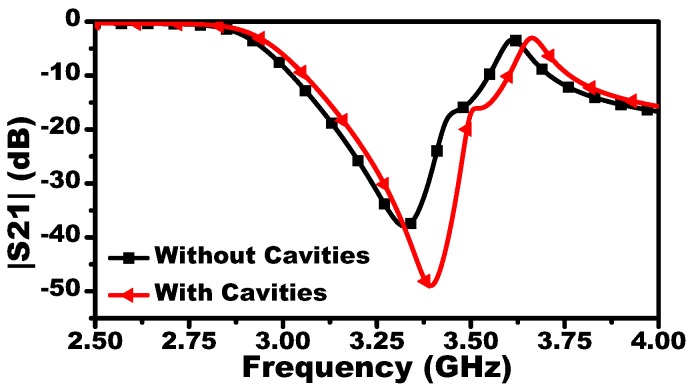
The scattering parameter $|S_{21}|$ dB of a three-resonator system with and without the aluminum cavities.

**Figure 5:**
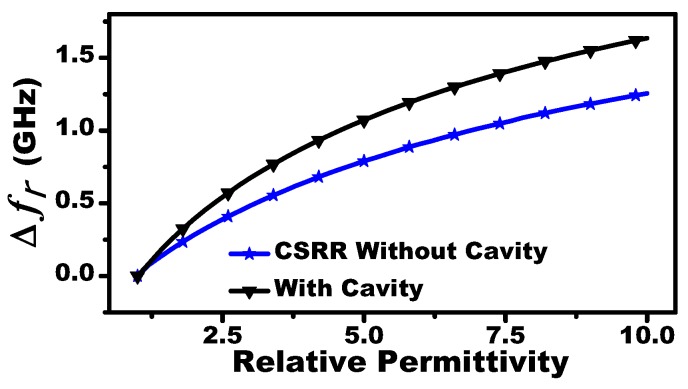
The sensitivity of a single CSRR (shift in the resonance frequency) versus relative permittivity of a dielectric slab with a thickness of 3 mm, with and without a cavity. The relative permittivity of a dielectric slab is varied from 1 to 10 with an increment of 0.2.

The MIS system will be tested by characterizing fluid concentrations. To this end, three-metallic cavities are added to contain the fluids. Figure 4 shows the frequency response of the MIS system with and without the metallic cavities. In fact, adding the cavities increased the sensitivity of the system as shown in Figure 5. This enhancement can be attributed to higher confinement of the electromagnetic field in the sensing area since the cavities are made of metallic material. The fluid concentration of a mixture material ($M_{m}$) is prepared by mixing two pure liquids: chloroform ($M_{m}$) and cyclohexane ($M_{l}$), with permittivities of 4.806 and 2.015, respectively (taken from [11]).

**Figure 6:**
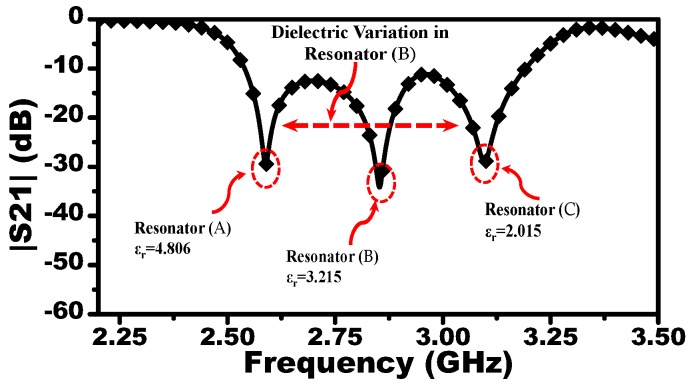
The scattering parameter $|S_{21}|$ of the microwave intelligent sensing (MIS) obtained using HFSS, where the resonators A and C are loaded by dielectric materials with the relative permittivity of 4.806 and 2.015, respectively, while resonator B is loaded by the dielectric material with permittivity of 3.215.

**Figure 7:**
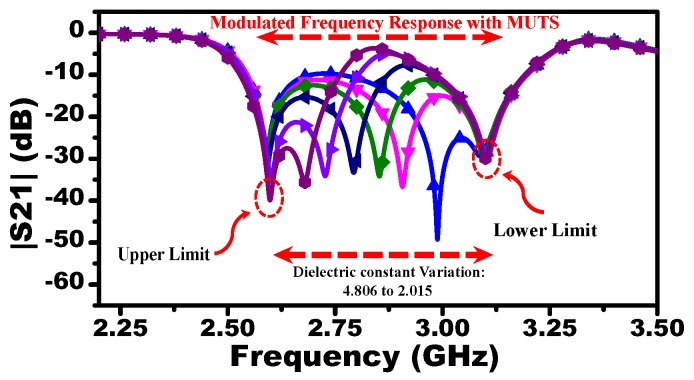
The scattering parameter $|S_{21}|$ of the system obtained from the full-wave numerical simulation. The resonators A and C are loaded with 4.806 and 2.015, respectively, while resonator B is loaded with material having permittivity varying from 2.015 to 4.806 in increments of 0.05 producing 57 curves. The plot is corresponding only to some selected variation for the purpose of making the curves clear.

To study the effect of different liquid concentrations, the resonators A and C are loaded by dielectric materials with relative permittivity of 4.806 and 2.015, respectively, while resonator B is loaded by a dielectric material with its permittivity varying from 2.015 to 4.806 with an increment of 0.01. Thus, the response to pure cyclohexane and chloroform will determine the upper and lower frequency limits, whereas the response of the mixture will be presented as a notch in the $|S_{21}|$ located between the two frequency limits. For instance, Figure 6 shows the response of the MIS where the resonator B is loaded with a material with relative permittivity of 2.015. Since $M_{u}$ has the highest dielectric constant, the resonance frequency of the resonator A will exhibit the highest shift with respect to free space (upper limit), whereas the resonance frequency of the resonator C loaded with $M_{l}$ will exhibit the lowest shift (lower limit). Thus, the response of the system interacting with the mixture ($M_{m}$) will vary between the upper and lower resonance frequencies. With the dielectric variation of $M_{m}$, the response of the system modulated with the reference materials ($M_{u}$) and ($M_{l}$) and the MUT ($M_{m}$) are extracted using the commercial three-dimensional full-wave simulation ANSYS HFSS [12] as shown in Figure 7.

**Figure 8:**
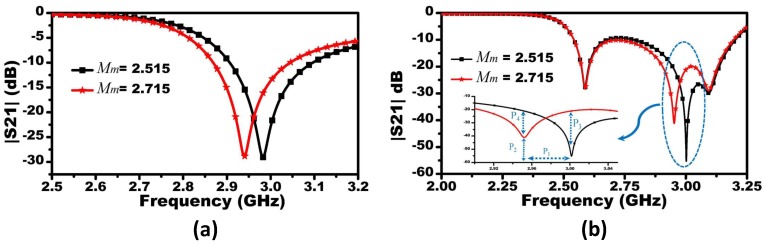
(**a**,**b**) The scattering parameter $|S_{21}|$ of the single CSRR and three CSRR based sensors, respectively, for $M_{m}$ having relative permittivity of 2.515 and 2.715.

The response of the MIS system was analyzed and compared to a single CSRR based systems. Both systems were tested to detect materials with dielectric constants of 2.515 and 2.715. The responses of the single-CSRR and three-CSRR sensors are shown in Figure 8a,b, respectively. To quantify the sensitivity enhancement of the proposed MIS sensing platform, four parameters, namely, $P_{1}$, $P_{2}$, $P_{3}$, and $P_{4}$ (see Figure 8b), were chosen to make the comparison between the two systems. $P_{1}$, $P_{2}$, $P_{3}$, and $P_{4}$ represent the shifts in the resonance frequency, changes in the $|S_{21}|$ at the two resonance frequencies (at the notch of the $|S_{21}|$ of the two systems), changes in the $|S_{21}|$ at the first resonance frequency (the presence of $M_{m}$ with the value of 2.515), and changes in the $|S_{21}|$ at the second resonance frequency (the presence of $M_{m}$ with the value of 2.515), respectively. For $P_{1}$, the sensitivity is enhanced by 7 MHz or equivalent to 16.5% when the shifts in the resonance frequencies (49.4 and 42.6 MHz) of the MIS system and the single CSRR based system, respectively, are compared. For $P_{2}$, when compared to the single CSRR based system, the sensitivity enhancement in terms of changes in the $|S_{21}|$ of the MIS system when the resonance frequency exhibits a change from 3 to 2.95 GHz, is 14 dB or equivalent to 78.21%. For $P_{3}$, the sensitivity enhancement that is evaluated at a single frequency (the resonance frequency of 3 GHz of the MIS system) based on the changes in $|S_{21}|$ is calculated as 21.24 dB or equivalent to 163.38%. Finally, $P_{4}$ shows a sensitivity enhancement of 6.38 dB or 46.73% at the resonance frequency of 2.95 GHz. This enhancement in the sensitivity indicates that the overall sensitivity of the proposed MIS system is enhanced over a wide range of frequencies. This enhancement of sensitivity is essential for increasing the accuracy of the entire detection system.

## 4. Fabrication and Experiential Results

**Figure 9:**
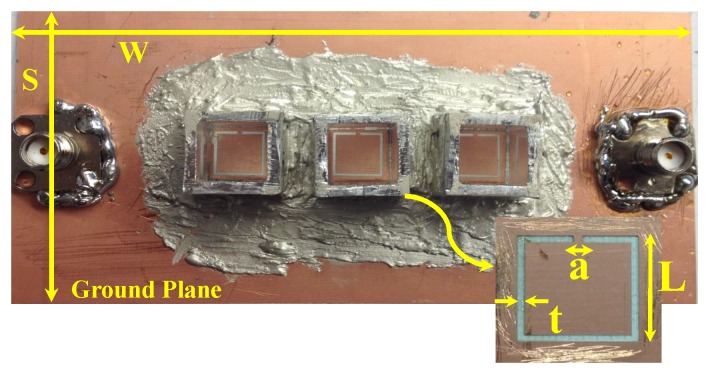
The fabricated MIS system, where L = 7.5 mm, t = a = 0.5 mm, W = 100 mm, and S = 50 mm.

The MIS was fabricated using printed circuit board (PCB) technology. Three aluminum cavities were added to the resonators to hold liquids. The sensing system is shown in Figure 9. Since the system will be tested to detect the concentration of a fluid material that is a mixture of chloroform and cyclohexane, the mixture was prepared where the chloroform was used as the reference material having a total liquid volume of 1 mL. For instance, 60% is corresponding to 0.6 mL of chloroform and 0.4 mL of cyclohexane.

**Figure 10:**
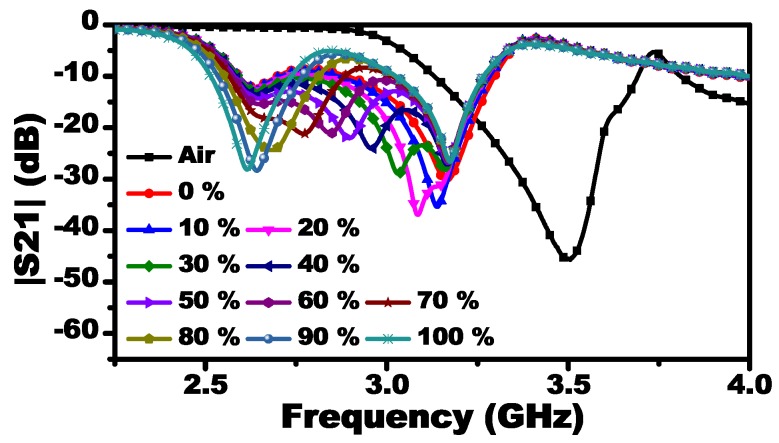
The response of the MIS obtained experimentally when using the chloroform as the reference material.

Twenty-one samples were prepared with different concentrations from 0% to 100% with an increment of 5%. The response of the MIS at each concentration is observed as a vector, $|S_{21}\left(f_{1},f_{2},\ldots ,f_{n}\right)|$ where $f_{1}$ = 2 GHz and $f_{n}$ = 4.50125 GHz with a step frequency value of 1.88 MHz. Each vector has 1335 components. Therefore, for 21 samples, we have 28,035 points of data to be processed. Since the concentration of the fluid is constant over these frequencies, which are going to be enforced by utilizing the supervised learning algorithm, the data points provide new information about the concentration. In fact, extracting more information about the targeted parameter can increase the accuracy of the results if the collected data has noise since the system will have different sensitivity over the range of frequency considered. For instance, the system might be sensitive to the air gap at a certain frequency but less sensitive at others. Thus, the system will be more robust at overcoming the sources of errors that are the result of the experimental setup. In addition, the information can be utilized to extract many parameters simultaneously. Note that, if we have a single input (a single frequency response at the resonance frequency), using 21 samples might not be sufficient to train the neural network. The responses of some selected concentrations are shown in Figure 10. For characterizing $M_{m}$ using the information contained in the samples, supervised neural networks can be used.

## 5. Demodulation Using Supervised Neural Networks

To find the relationship between the input and the output (the liquid concentration in our case), the multi-layer perceptron (MLP) neural networks with supervisor learning algorithms can be utilized. There are many backpropagation learning algorithms such as Bayesian regulation backpropagation, Levenberg–Marquardt backpropagation, scaled conjugate gradient backpropagation, and resilient backpropagation. Since the datasets are small and limited to only 21 samples, the Bayesian regulation backpropagation algorithm is the best choice to use for the learning process of the neural networks [13]. The Bayesian regulation backpropagation artificial neural network (BRANN) can produce robust models without the need of the validation process that is required by other algorithms that need some of the data for validation [13]. In addition, the BRANN overcomes the difficulty of overtraining and overfitting.

The BRANN was tested with different hidden layers since higher number of hidden layers are not necessary to produce higher accuracy. Four and five hidden layers were found to be sufficient for our datasets. In the BRANN, the datasets are divided into training and test (unseen) samples. In particular, the datasets were classified as 1—70/100% training 30/100 test (14 samples for training and six samples for the test) 2—85/100% training 15/100 test (18 samples for training and three samples for the test) 3—90/100% training 20/100 test (19 samples for training and two samples for the test). In the supervisor learning algorithm, the choice of particular samples for training or testing is random to test the performance of the BRANN. Since the datasets are small, we found that training the BRANN with 19 samples can result in an accuracy of approximately 99.9%. The summary of the training and the test samples with different hidden layers and classification of the datasets and the performance in the mean square error (MSE) is presented in Table 1.

## 6. Conclusions

This paper introduced a microwave intelligent sensing systems. The system is based on modulating and demodulating the frequency response of multiple closely distanced sensors over a wide frequency range. Under the assumption that the physical parameters to be characterized such as fluid concentration are constant over the frequency band of the sensor, the responses characterized as vectors of multiple dimensions of the transmission coefficients are then utilized for constructing and testing 205 models that were built using neural network techniques. Furthermore, to increase the sensitivity of the system over a larger frequency band, multiple identical electrically-small resonators are used. In this work, three identical CSRRs were used. The system was tested to detect the concentration of fluids. The sensing part of the system was fabricated using PCB technology, and the results were recorded using a vector network analyzer. Then, the results were used to construct models utilizing the Bayesian regulation backpropagation artificial neural network (BRANN). The models were used to characterize the fluids. Although the datasets were relatively small, the utilization of the vectors of many components as inputs provided sufficient information to the BRANN to produce models with an accuracy ranging from 82.8% to 99.97%. An accuracy level of 99.97% was achieved as a result of using 90% of the data to train the BRANN, which shows that larger datasets provide higher accuracy. 

## Figures and Tables

**Table 1:** The Bayesian regulation backpropagation artificial neural network (BRANN) for characterizing the concentration of a fluid material.

Training Trial	Hidden Layers	Training to Test Samples	Training Performance in Mean Sqaure Error (MSE)	Overall Performance MSE	Test Performance in MSE	Unseen Results (Test)	Results Using the BRANN	Accuracy
1	5	15 to 6	1.0578 × 10${}^{-20}$	0.6179	2.1627	10 20 30 35 50 95	11.73 21.2172 28.5424 35.9368 52.0599 96.1383	82.80% 93.90% 95.00% 97.00% 95.80% 98.80%
2	5	18 to 3	0.2837	0.0017	1.9754	20 35 40	20.0066 35.0105 40.4515	99.90% 99.90% 98.80%
3	5	18 to 3	0.0017	0.1474	1.022	20 70 75	20.18 70.59 76.64	99.10% 99.20% 97.80%
4	5	18 to 3	3.3052 × 10${}^{-15}$	0.2153	1.5074	75 80 90	75.14 78.42 88.58	99.80% 98.00% 98.00%
5	5	18 to 3	7.494 × 10${}^{-4}$	0.1572	1.0962	45 50 65	45.61 51.72 65.4	99.10% 96.60% 99.40%
6	5	19 to 2	1.6865 × 10${}^{-4}$	0.0539	0.5648	25 55	25.59 54.12	98.40% 97.64%
7	4	19 to 2	2.7125 × 10${}^{-24}$	0.2888	3.0322	20 100	20.4 97.57	98.00% 97.60%
8	4	19 to 2	3.6725 × 10${}^{-4}$	3.6155 × 10${}^{-4}$	3.0743 × 10${}^{-4}$	40 60	39.99 59.98	99.97% 99.96%
9	4	19 to 2	1.4986 × 10${}^{-13}$	0.1053	1.106	20 90	19.6 88.57	98.00% 98.00%
10	4	19 to 2	1.2156 × 10${}^{-13}$	0.0718	0.7539	55 95	54.2 95.93	98.50% 99.00%
11	4	19 to 2	0.002	0.0761	0.7803	40 75	40.08 76.25	99.80% 98.00%
12	4	19 to 2	1.9130 × 10${}^{-14}$	0.0943	0.9906	15 60	13.59 59.97	90.60% 99.60%
